# Knowledge of preconception care and its association with family planning utilization among women in Ethiopia: meta-analysis

**DOI:** 10.1038/s41598-021-89819-8

**Published:** 2021-05-25

**Authors:** Addisu Alehegn Alemu, Mezinew Sintayehu Bitew, Liknaw Bewket Zeleke, Yewbmirt Sharew, Melaku Desta, Ermias Sahile, Yayehyirad Yemaneh, Getachew Mullu Kassa

**Affiliations:** 1 College of Health Sciences, Debre Markos University, P.O.BOX: 269, Debre Markos, Ethiopia; 2 College of Health Sciences, Kotebe Metro Politan University, Addis Ababa, Ethiopia; 3 SRH Program Manager International Medical Corps, Afar Region, Ethiopia

**Keywords:** Epidemiology, Outcomes research, Paediatric research, Preclinical research, Biochemistry, Cancer, Endocrinology, Health care, Medical research, Risk factors

## Abstract

Preconception care (PCC) increases the chance of couple’s being healthy and having a healthier baby. It is an important strategy to prevent maternal and perinatal complications. The level of knowledge on preconception care increases its uptake. It is also considered as an input for further intervention of reduction in maternal and neonatal mortality enabling progress towards sustainable development goals (SDGs). Therefore, this systematic review and meta-analysis aimed to estimate the pooled knowledge level of PCC and its association with family planning usage among women in Ethiopia. All observational studies regardless of publication status were retrieved. Important search terms were used to search articles in Google scholar, African Journals Online, CINHAL, HINARI, Science Direct, Cochrane Library, EMBASE, and PubMed/Medline. Independent critical appraisal of retrieved studies was done using the Newcastle–Ottawa assessment checklist. The meta-analysis was conducted using STATA version 14 software. The I^2^ statistics were used to test heterogeneity, whereas publication bias was assessed by Begg’s and Egger’s tests. The results of the meta-analysis were explained in the Odds ratio (OR) with a 95% confidence interval (CI) and presented using forest plots. A total of seven articles were included in the current systematic review and meta-analysis. Based on the data retrieved from the articles, 35.7% of women in Ethiopia had good knowledge about preconception care. The subgroup analysis based on region revealed the lowest (22.34%) and highest (45.06%) percentage of good knowledge on preconception care among women who were living in Amhara and Oromia regions, respectively. Moreover, women who utilized family planning services were three and more times (OR 3.65 (95% CI 2.11, 6.31)) more likely to have a good level of knowledge about preconception care. One-third of Ethiopian women had good knowledge about preconception care. Family planning utilization had a positive impact on women’s knowledge of preconception care.

## Introduction

Preconception care (PCC) is an important and preventive health care intervention for couples before conception^1,2^. PCC is an interventional opportunity to improve maternal outcomes and the future generation^3,4^. It is a strategy by which biomedical, behavioral and social health-related interventions are provided to the couples through risk screening and health education^5–7^. It also considers treatments, if indicated^2^. Generally, PCC is a cost-effective tool for women with chronic disease in the primary setting^8^.

PCC contributes to reducing maternal and childhood mortality and morbidity, globally^9^. Its role outweighs in pockets of socially marginalized and economically deprived families and communities^8,9^. It reduces potential low birth weight, abortion, prematurity, and congenital anomalies, and maternal hyperglycemia^10–13^. Its effective utilization also improves maternal and child health through the early initiation of antenatal care^10,14^. Moreover, pregnant women who received PCC were more likely to be supplemented with foliate, be vaccinated, received a better level of care for their pre-existing health condition^15^. Because of the above reasons, the majority of maternal and infant morbidities and mortalities are reduced through quality PCC^16,17^.

Although there are appropriate interventional strategies to increase the knowledge and uptake of PCC in the community^18,19^, the current studies revealed women’s awareness about PCC and its utilization is low^20,21^. The knowledge level of PCC varies significantly from across the world^22,23^. Studies were done on knowledge on preconception care found between 17.3%^16^ in Ethiopia and 71.9% in Lebanon^22^. Nowadays clinical interventions to be offered before conception to prevent adverse pregnancy outcomes have been identified^24^. But, the practice and implementation of preconception care are still at their initial phase^25^. Due to the low level of knowledge on PCC, its utilization is also lower^20,26^. Likewise, the couple’s intention to seek out PCC is insufficient^23^. Different studies in Ethiopia showed the women’s knowledge of PCC as a basic issue for implementation of a maternal continuum of care^27–29^ However, none of them are inconclusive in determining the knowledge level and its predictors.

The national-level estimation is vital to design and apply evidence-based strategies. Especially in the less developed world where maternity care is started after the second half of pregnancy age^30^.

Therefore, this meta-analysis was conducted to know the national level knowledge of preconception care among women in Ethiopia. The review question was what is the knowledge and utilization of preconception care among pregnant women in Ethiopia?

## Methods

### Search strategy and study selection

We conducted this systematic review and meta-analysis of all observational published studies to assess the pooled prevalence and determinants of preconception care in Ethiopia. Retrieving of the included studies was done in different databases such as Google scholar, African Journals Online, CINHAL, HINARI, Science Direct, Cochrane Library, EMBASE, and PubMed (Medline). Moreover, grey literature was considered through reviewing the lists of references. Additionally, unpublished work eligible for our meta-analysis in Addis Ababa digital library was searched. The Preferred Reporting Items for Systematic Reviews and Meta-analysis (PRISMA) guideline was strictly followed during systematic review and meta-analysis^31^.

A combination of search terms that best describe the study variables were used to retrieve articles. These include risk factors, determinants, predictors, factors, magnitude, prevalence, incidence, preconception care, knowledge, and Ethiopia. The terms were combined using “OR” and “AND” Boolean operators. Additionally, the reference list of the already identified articles was checked to find additional eligible articles but was missed during the initial search. The searching was carried out from June 2 to July 1, 2020.

### Eligibility criteria

#### Inclusion criteria

All observational published and unpublished studies that reported knowledge of preconception care among pregnant women in Ethiopia were included.

#### Exclusion criteria

Clinical trials, case reports, ecological studies, and reviews were excluded. Moreover, articles that were not fully accessible were excluded after a minimum of three email contacts with the cross ponding authors.

### Outcome of interest

The main outcome of interest was the prevalence of knowledgeable women on PCC in Ethiopia. The prevalence of knowledgeable women was computed based on the correct response using PCC knowledge questions^32^. Estimating the association between PCC knowledge and family planning usage was the second outcome of interest.

### Data extraction

Three authors (AAA, LBZ & GMK) searched all records independently then extracted the data using standardized format on Microsoft Excel. The format included: first author, publication year, study design, region of study, sample size, knowledge of PCC, and study's quality. Finally, the other authors (MSB, ES, YS, YY & MD) were involved in resolving the disagreements on the quality score of each study.

### Quality assessment of studies

Each record included in this meta-analysis was assessed by the two authors (AAA & GMK), intensively using the Newcastle–Ottawa assessment checklist for observational studies^33^. The checklist has three parts; the methodological assessment and the comparability evaluation are rated up to five and three stars respectively. Similarly, the important variables including the outcome variable were taken and used in this study. Articles scored ≥ 6 out of 10 were considered as high quality and utilized for this meta-analysis. The uncertainty between the assessors was solved with discussion considering the score by another author.

### Data synthesis and statistical analysis

The data were exported from Microsoft Excel to STATA version 14 software^34^ for statistical analysis. The summarized and descriptive results were shown using figures and tables. This review was conducted to determine the knowledge and utilization of preconception care in Ethiopia. The pooled knowledge of preconception care among reproductive pregnant women was assessed considering the random-effect model. Due to the heterogeneity by study design and study regions /areas. *I*^2^ statistics of 25, 50, and 75% were used to declare low, moderate, and high heterogeneity, respectively^35^. We had subgroup analysis by region because of the heterogeneity among the included studies to estimate the pooled prevalence. We have also checked publication bias using Egger’s and Begg’s tests, and a p-value of less than 0.05 was used to declare its statistical significance^36,37^.

## Results

### Study selection

**Figure 1:**
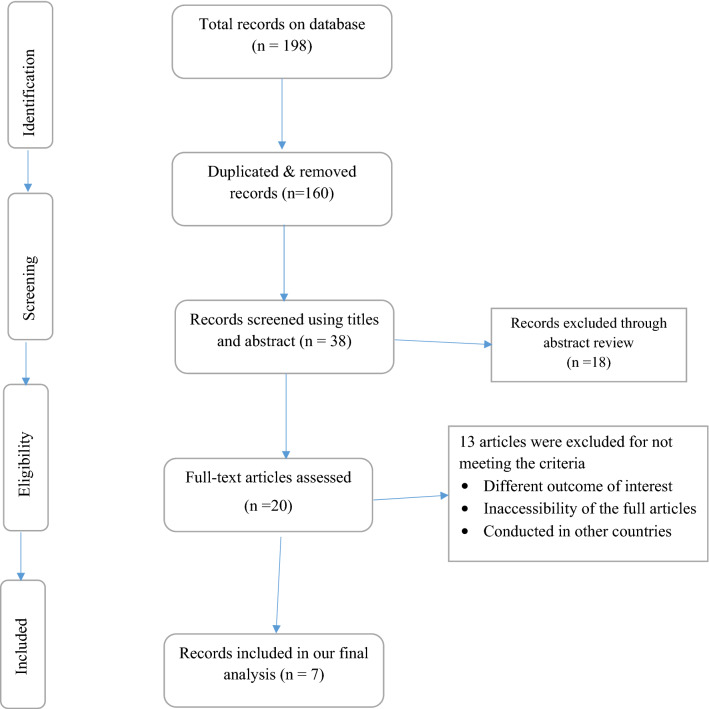
Flow chart showing records considered for systematic and meta-analysis of family planning usage and its association with knowledge of preconception care among women in Ethiopia.

All observational studies on preconception care among women in Ethiopia were included in this systematic and meta-analysis. A total of 198 articles were found on the databases 160 of which were duplicated and removed through title screening. After a screening of all the retrieved records, 18 articles were excluded by reviewing their abstracts. A total of 20 full-text articles were assessed for eligibility, finally, 7 studies were included in the meta-analysis of this study (Fig. 1).

### Characteristics of included studies

A total of seven observational studies^16,17,32,38–41^ were included in the current systematic and meta-analysis. The studies were both institution and community-based, reported knowledge of preconception care. Of the included studies^16,32^, were conducted in Amhara^38,39^, were conducted in Oromia^17,41^, were conducted in SNNPR, and^40^ was conducted in Addis Ababa (AA). The sample size of included studies ranged from 142 in AA^40^ to 669 in Oromia^39^. A total of 2995 women participated in the current study. The included studies’ quality was between 6 and 9 (Table 1).

**Table 1:** Characteristics of studies included in the final meta-analysis.

First author	Year of publication	Region	Sample size	Knowledgeable (%)	Study design	Quality
Demisse et al.^16^	2019	Amhara	410	17.3	Community based cross sectional	9
Fekene et al.^39^	2020	Oromia	669	26.8	Community based cross sectional	9
Gezahegn^38^	2016	Oromia	402	63.4	Institution based cross sectional	6
Kassie^40^	2018	AA	142	42.7	Institution based cross sectional	6
Yohannes^41^	2019	SNNPR	370	53	Institution based cross sectional	7
Kassa and Yohannes^17^	2018	SNNPR	580	20	Institution based cross sectional	8
Ayalew et al.^32^	2017	Amhara	422	27.5	Community based cross sectional	9

### Knowledge of preconception care among women in Ethiopia

**Figure 2:**
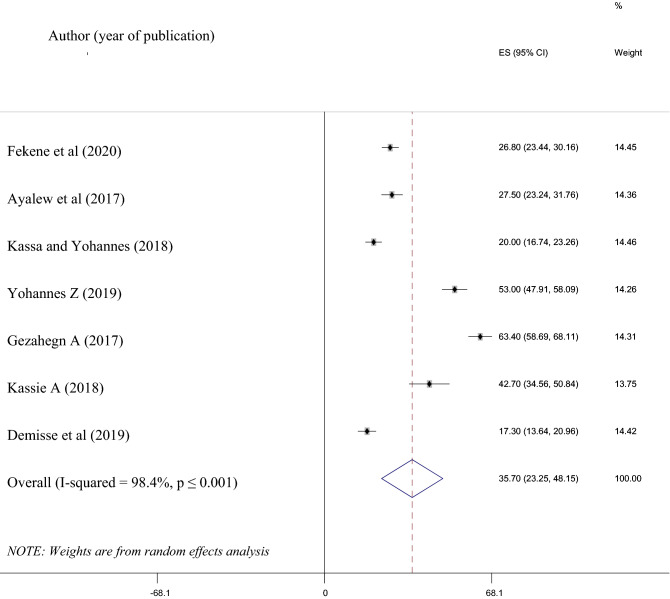
Shows the pooled estimated knowledgeable women about preconception care in Ethiopia.

The women’s knowledge on PCC among the studies included in this study was ranged from 17.3% in Amhara^16^ to 63.4% in Oromia^38^. Due to the high heterogeneity (*I*^2^ = 98.4%) observed in this analysis, the random effect model was considered to estimate the pooled prevalence. Therefore, the pooled estimated knowledgeable women in Ethiopia on preconception care was 35.70% (95% CI 23.25, 48.15) (Fig. 2).

### Subgroup analysis

**Figure 3:**
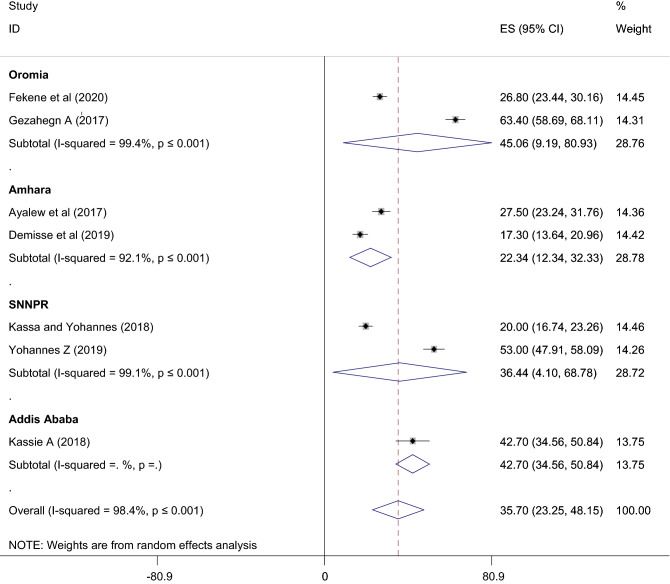
Preconception care knowledgeable women by region in Ethiopia.

Subgroup analysis by region was conducted due to the high heterogeneity observed. Based on this, the highest knowledgeable women on PCC were found in Oromia 45.06% (95% CI 9.19, 80.93). Whereas, the lowest knowledgeable women were found in Amhara 22.34% (95% CI 12.34, 32.33). Moreover, the Duval and filled analysis was conducted to fill the publication bias identified by Egger test with unpublished studies (Fig. 3).

### The association between family planning usage and PCC knowledge

**Figure 4:**
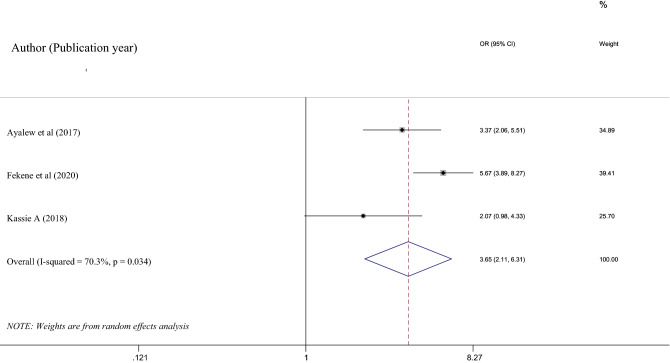
Shows the effects of family planning utilization on women’s preconception knowledge in Ethiopia.

Three studies^32,39,40^ that fulfilled the inclusion criteria were included to determine the association of pooled PCC knowledge and family planning usage among women in Ethiopia. All of the studies showed a history of family planning usage had a positive association with PCC knowledge. Due to the moderate heterogeneity (*I*^2^ = 70.3%) observed in this analysis, a random effect meta-analysis model was employed to determine the association. Based on this, family planning usage had a significant association with PCC knowledge 3.65% (95% CI 2.11, 6.31), p-value = 0.034. Moreover, the absence of potential publication bias was confirmed with a p value of 0.165 and 0.296 Egger’ test and Begg’s test, respectively (Fig. 4).

## Discussion

Every reproductive-aged woman should receive preconception care before she becomes pregnant^42^. PCC is an important means of improving women’s and infants’ health outcomes^43^. To have improved PCC multi-strategic interventions are required^44^. It needs serious attention from the government and stakeholders^27^. The current systematic review and meta-analysis were aimed to identify the knowledge of PCC and its association with family planning usage among women in Ethiopia.

This study disclosed the pooled knowledgeable pregnant women in Ethiopia on preconception care were 35.70% (95% CI 23.25, 48.15). This finding is consistent with the other studies conducted in Turkey (46.3%)^45^ and Saudi Arabia (37.9%)^46^. But, it is higher than the findings from studies conducted in Sudan (11.1%)^47^ and Iran (10.4%)^48^. The possible reason for the observed difference might be due to the difference in study participants and methods of assessment. The study in Sudan was conducted exclusively among reproductive-aged women with rheumatic heart disease, unlike the current study that considered all reproductive-aged women. Unlike the articles included in the current meta-analysis, the Donabedian model utilized for the study in Iran could bring the difference.

However, the magnitude of knowledgeable women in this meta-analysis is lower than the study conducted in Malaysia which showed 51.9% of women were knowledgeable about PCC^49^. Similarly, the finding from the current meta-analysis is lower than studies conducted in Saudi Arabia, the United States of America, and Jordan revealed that (84.6%), (76%) and (85%), respectively^50–52^. The difference in socio-demographic characteristics, sampling, and study setting of study participants, the studies considered might the possible reasons for the discrepancy observed. The study from Malaysia included educated women whereas the current meta-analysis women regardless of their educational status. It is evident education improves the skill of searching information^32^ and maternal awareness of her health care services^53^. Likewise, for the studies in Saudi Arabia and the U.S.A, a non-probability sampling method was employed to select the participant. Moreover, the studies in U.S.A and Jordan were conducted exclusively in maternal and child care clinics unlike the current meta-analysis included both institution-based and community-based studies.

The utilization of family planning among study participants in this meta-analysis is significantly associated with knowledge on PCC. Women who utilized fa2mily planning services were more likely to be knowledgeable about PCC. This finding is supported by the studies conducted in Sudan^54^. This might be due to family planning service is a means to improve individuals’ health condition before a planned pregnancy^55^. Women who postpone their pregnancy have an increased need for preconception counseling^55^. Therefore, the prevalent neural tube defect in Ethiopia^24^ can be prevented through preconception counseling during family planning service provision.

### Limitations of the study

This meta-analysis has limitations. Only articles and reports in the English language were included. Additionally, all the included articles were cross-sectional study design, in which the association might be affected by confounders. Moreover, the included studies were from only two regions and one city administrative. Therefore, the findings from this meta-analysis may not be representative of the country due to the limited number of articles.

## Conclusions

This meta-analysis revealed, less than two-fifth of reproductive-aged women are knowledgeable about PCC in Ethiopia. Likewise, family planning utilization has a significant association with PCC knowledge.

## Data Availability

Data used for this study are available from the authors of each included study upon reasonable request.

## Abbreviations

PCC: Preconception care
AA: Addis Ababa
SNNPR: South Nations Nationalities and People’s Region
PRISMA: Preferred Reporting Items for Systematic Reviews and Meta-analysis
CI: Confidence interval
AOR: Adjusted odds ratio
